# Effects of Physical Exercises in Asthma: An Umbrella Review of Systematic Review and Meta‐Analysis

**DOI:** 10.1111/crj.70075

**Published:** 2025-04-17

**Authors:** Fuchun Huang, Shuang Yang, Mingxuan Ma, Jialin Zhang, Hua Liu

**Affiliations:** ^1^ Department of Tuberculosis Hangzhou Red Cross Hospital Hangzhou China; ^2^ Medical School of Nantong University Nantong Jiangsu China; ^3^ Department of Respiratory and Critical Care Medicine Affiliated Hospital of Nantong University Nantong Jiangsu China

**Keywords:** asthma, meta‐analysis, physical exercise, systematic review, umbrella review

## Abstract

**Background:**

As a disease‐modifying strategy, physical exercise has been demonstrated to significantly improve quality of life, exercise capacity, and lung function in individuals with asthma. However, the quality and robustness of this evidence has not been thoroughly examined in many large‐scale investigations. In order to assess the evidence addressing the effects of physical exercise in patients with asthma, we therefore carried out an umbrella review.

**Methods:**

A comprehensive search of the PubMed, Web of Science, and Cochrane databases for systematic reviews and meta‐analyses of the effects of physical activity on asthma was conducted up to December 31, 2022. The study was registered in Prospero (CRD42023382921). For every qualified systematic review and meta‐analysis, we extracted information on the main characteristics and general findings. The GRADE tool was utilized to quantify the strength of the evidence, and the AMSTAR2 score was employed to evaluate the methodological quality.

**Results:**

A total of 1254 articles were searched, and 42 independent results were identified as eligible in the 11 articles that were included. Of the 42 unique outcomes, two were rated as high, two as medium, and the rest as low or very low. Physical exercise is beneficial for improving QoL, exercise capacity, and lung function in patients with asthma.

**Conclusions:**

According to our research, physical activity benefits QoL of patients with asthma, exercise tolerance, and lung function. In the future, further evidence from superior prospective studies will be required because the quality of the available evidence is now insufficient.

## Graphical abstract

Physical activity benefits QoL of patients with asthma, exercise tolerance, and lung function. But further evidence from superior prospective studies will be required because the quality of the available evidence is now insufficient.

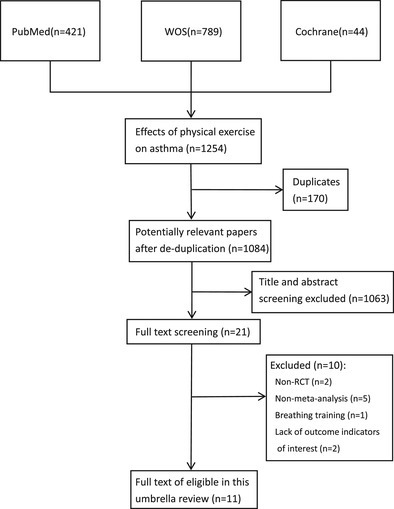

## 1 Introduction

Bronchial asthma is a common chronic inflammatory disease of the airway. The prevalence of asthma is still increasing in different countries in recent years, seriously endangering human health and quality of life (QoL) [1, 2]. Although a wide range of medications are available for the relief and control of asthma, the results of treatment in some populations are unsatisfactory, and the toxic side effects caused by long‐term use of medications are difficult for us to avoid [3, 4].

With the intensive study of asthma, physical exercise has attracted much attention from humans as an alternative treatment for asthma. The GINA encourages children with asthma to participate in appropriate physical activities. Research has shown that people with asthma can safely participate in physical activity under medical supervision and management [5]. To a certain extent, the appropriate intensity of physical activity can improve QoL, exercise capacity, and lung function in people with asthma [6, 7]. It can also reduce dependence on glucocorticoids [8]. A randomized controlled trial (RCT) by Scholar Andrade [9] concluded that aerobic exercise interventions were significantly better than non‐exercise interventions in improving QoL and exercise capacity in patients with asthma. In a Norwegian population‐based cohort study [10], it was found that physically active participants had a slightly smaller annual decline in lung function than inactive asthmatic participants.

The effects of physical activity on patients with asthma have been summarized by a sizable number of meta‐analyses to date; however, the quality of the evidence differs significantly between research. Simultaneously, there have not been many thorough investigations of the reliability and validity of the evidence. The umbrella review compiles all available meta‐analyses evidence and ranks it based on predetermined standards. It measures the strength and viability of the evidence as well as the degree of bias [11]. We therefore conducted a comprehensive evaluation of the most recent data from existing systematic reviews and meta‐analyses in order to properly assess the evidence that is currently available on QoL, exercise capacity, and lung function in individuals with asthma.

## 2 Methods

### 2.1 Study Design

The umbrella review is a compilation of previous systematic reviews and meta‐analyses created to compile data from several studies on a specific subject [11, 12]. We conducted this umbrella review to assess the available evidence on QoL, exercise capacity, and lung function between physical activity and patients with asthma.

The Preferred Reporting Items for Systematic Evaluation and Meta‐Analyses 2020 (PRISMA 2020) statement criteria were followed in conducting this umbrella review [13]. The umbrella review protocol was submitted to PROSPERO (registration number: CRD42023382921).

### 2.2 Literature Search Strategy

Fuchun Huang and Shuang Yang, two of the writers, separately carried out a thorough literature search utilizing the Cochrane, Web of Science, and PubMed databases. In order to find systematic reviews and meta‐analyses of RCTs, we looked for articles that had been published between the creation of the database and December 31, 2022.

The search algorithm uses the following terms/keywords:

- (endurance OR strength OR exercise OR training OR sport OR physical activity OR run OR cycle OR swim OR gymnastics OR aerobic exercise OR anaerobic exercise) AND (asthma OR wheezing OR bronchial asthma) AND (meta‐analysis OR systematic review OR systematic overview).

There were no limitations on the study participants' ages or the language of publication. In order to find additional studies pertinent to our omnibus review, manual searches were also done on the reference lists of the publications that had been identified, references from previous omnibus reviews that dealt with physical activity, and research registry platforms. Discussions between the two authors helped to settle disagreements. Details of the search strategy and results can be found in Additional file 1: Table S1.

### 2.3 Selection and Exclusion Criteria

All titles and abstracts for the computerized search were independently screened for potential relevance by two investigators (Fuchun Huang and Shuang Yang).

The inclusion criteria were as follows: (1) meta‐analyses and systematic reviews of RCTs in line with PRISMA guidelines; (2) systematic reviews and meta‐analyses to evaluate the relationship between physical activity and patients with asthma: the study subjects met the diagnostic criteria for asthma, and the interventions included aerobic training (swimming, ball games, jumping rope, jogging, cycling), strength training, and balance and coordination training, and the outcome indices included at least one or more of the QoL, Exercise Capacity (V02max), or Lung Function (FEV1, FEV1%Pred, FVC, FVC%, PEF, PEF%); (3) no participants and language restrictions were used in the selection of eligible studies.

The exclusion criteria were as follows: (1) animal studies; (2) narrative reviews, primary research, conference proceedings, and letters to the editor; (3) full‐text literature that was not available through a variety of sources and methods; (4) studies that simply performed respiratory muscle training, breathing exercises, pulmonary rehabilitation, or yoga; (5) studies with missing data: MD, SMD, and their corresponding 95% confidence interval (CI).

### 2.4 Data Extraction

Two authors (Fuchun Huang and Shuang Yang) extracted the data separately.

The third author, Mingxuan Ma, reevaluated any discrepancies in the extracted data. We first collected the following common traits for every valid systematic review and meta‐analysis: (1) the first author; (2) the year of publication; (3) original article retrieval time; (4) the journal; (5) the type of physical activity in the study; (6) the number of included studies; (7) the findings of interest (QoL, exercise capacity, lung function), the country or region of the original study, and the number of corresponding studies; (8) the type of design of the included original study (randomized controlled study); (9) the number of participants in the study; (10) the quality assessment of the systematic evaluations or meta‐analyses of each of the eligible studies.

In addition, the key findings of each study were also distilled: (1) type of effect model; (2) meta‐analysis metric; (3) estimated pooled effects (MD, SMD), 95% confidence intervals (CIs) for estimated pooled effects, and *p*‐values for the tests; (4) heterogeneity (I2) and *p*‐values; (5) publication bias by Egger's (or Begg's) tests and small study effects.

### 2.5 Quality Evaluation

Meta‐analyses eligible for full‐text screening were conducted by one researcher (Mingxuan Ma) using the AMSTAR2 [14]. AMSTAR2 is a methodological quality appraisal tool with 16 items [14]. Of these, Items 2, 4, 7, 9, 11, 13, and 15 are critical items. The details are as follows: Q2: Did the protocol be established prior to conducting the review? Q4: Did a comprehensive literature search strategy be used? Q7: Did the excluded studies justify it? Q9: Did the risk of bias in the individual studies be fully assessed? Q11: Did meta‐analyses authors use the appropriate statistical methods? Q13: Did the risk of bias be adequately considered when discussing the review results? Q15: Did publication bias and its impact have been fully assessed?

Based on the AMSTAR2 scale, the methodological quality of the literature can be rated as follows:

- High: No or one non‐critical weakness [14].
- Moderate: More than one non‐critical weakness [14].
- Low: One critical flaw with or without non‐critical weaknesses [14].
- Critically low: More than one critical flaw with or without critical weaknesses [14].

In addition, we used the GRADE [15, 16] (Grading of Recommendations Assessment, Development and Evaluation GRADE) to evaluate the strength of the evidence in each meta‐analysis.

Comprehensive evaluation of evidence through a combination of five factors that decrease the quality of evidence (risk of bias, indirectness, inconsistency, imprecision, and publication bias) and three factors that increase the quality of evidence (large effects, dose–response gradient, and plausible confounding), the GRADE categorizes the quality of evidence into four levels: “High”, “Moderate”, “Low”, and “Very low”.

### 2.6 Statistical Analysis

We abstracted exposure, outcome, and the estimated summary effect (MD: Mean Difference, WMD: Weighted Mean Difference, SMD: Standardized Mean Difference) with their matching 95% CI and *p*‐value for each meta‐analysis that was included in our study. To measure study heterogeneity, Cochran's Q test and the I^2^ metric were applied. The chosen meta‐analyses choose random or fixed effect models from the original models. However, we did not perform a secondary analysis. Funnel plots and Egger's (or Begg's) tests were used to detect small effects. Funnel plot asymmetry or Egger's (or Begg's) test *p*‐value < 0.10 were considered evidence of a small‐study effect [17]. For the statistical tests of clinical outcomes, the significance threshold was set at *p* < 0.05.

## 3 Results

### 3.1 Characteristics of the Included Meta‐Analyses

**FIGURE 1:**
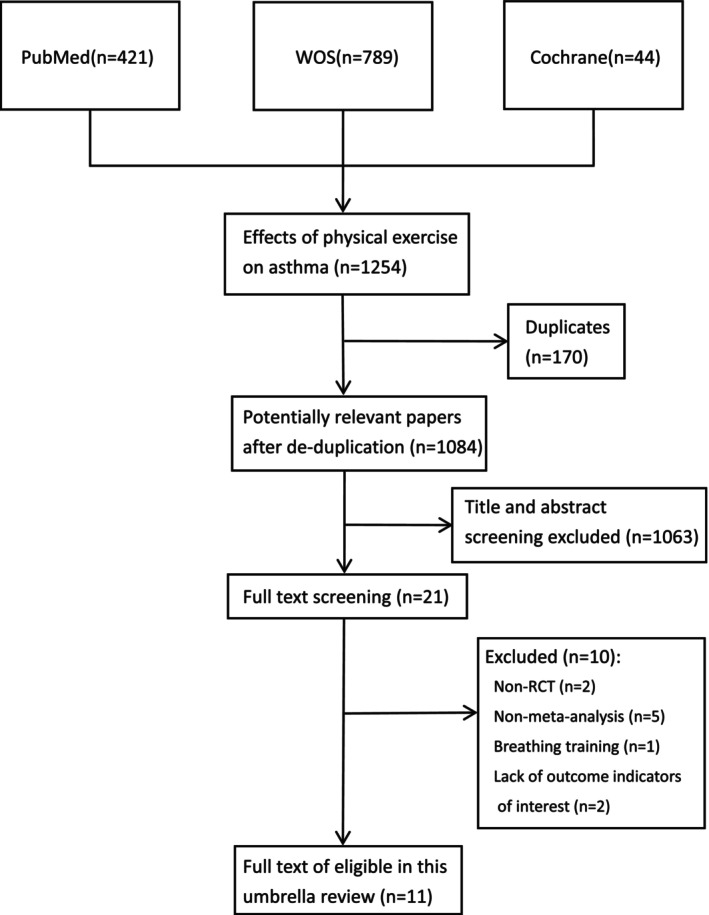
Literature screening process of this study.

The literature screening process is illustrated in Figure 1. Two authors independently and systematically searched 1254 papers with 170 duplicates, and the titles and abstracts of the remaining 1084 papers were reviewed for eligibility. Of these, 1063 studies were not relevant to the topic of the current study, and 21 studies were considered relevant. Of these studies, five did not perform a meta‐analysis, two included original studies that were not RCTs, one intervention was a respiratory training program, and two were unable to obtain outcome metrics of interest, resulting in the inclusion of 11 articles that met the eligibility criteria. A list of studies excluded after full‐text screening is provided in Additional file 2: Table S2.

The characteristics of the 11 included articles are listed in Table 1. Of the included articles, two did not have a methodological quality assessment of the original study [18, 19]. One study used the Jadad scale score [20] and Cochrane Handbook for Systematic Reviews of Intervention Criteria to assess risk of bias [21]. The remaining eight papers [22, 23, 24, 25, 26, 27, 28, 29] were assessed for risk of bias using the Cochrane Collaboration tool [30]. All the systematic reviews and meta‐analyses were published between 2012 and 2022. The original studies included in these studies were all randomized controlled studies, and the number of original studies included ranged from four to 22. These primary studies were conducted in the UK, the USA, France, China, Brazil, and Australia. The included studies covered a variety of exercise modalities such as aerobic exercise, strength training, and fixed treadmill exercise. The maximum number of participants in these studies was 1356, with 703 participants in the intervention group and 653 in the control group. The minimum number of participants was 217, with 123 participants in the intervention group and 94 in the control group.

**TABLE 1:** The general characteristics of the 11 systematic reviews and meta‐analyses.

First author, yea	Original article retrieval time	Journal	Sports type	No. of studies included in this review related to our topic	Continent/region/country; no. of studies	Type of studies	Study design (number)	Sample size	Quality assessment
Beggs S, 2013	Up to July 2012	Cochrane	Swimming (8)	8 studies	Turkey (1); Japan (1); France (2); Taiwan (1); American (2); Brazil (1)	Systematic review and meta‐analysis	RCT(8)	262 participants	Cochrane
Hadassah, 2021	Up to December 2020	International Journal of Nursing Studies	Swimming (9)	9 studies	Ireland (1); Taiwan (1); American (2); Japan (1); France (1); Australia (1); Brazil (1); Sweden (1)	Systematic review and meta‐analysis	RCT(5)	NA	Cochrane
Philipp, 2013	Up to November 2012	Sports Med	Swimming (7); running (5); aerobic exercise (2); cycling (1); aerobic exercise and strengthening (1); swimming and strengthening aerobic exercise (1);	17 studies	NA	Systematic review and meta‐analysis	RCT(17)	Intervention = 312 Control = 284	Cochrane
Xionghui, 2022	Between January 2000 and June 2021	Computational and Mathematical Methods in Medicine	Bicycle kick (2); Basketball (1); Taijiquan activity (1); Swimming (3); Setting up exercise (4); Gym strength training (1); Rope skipping, jogging (1);	13 studies	NA	Systematic review and meta‐analysis	RCT(13)	Experimental = 496 Control = 462	NA
Xinggui, 2020	Between 1990 and 2019	Journal of Thoracic Disease	Cycling and jogging (1); Swimming (4); Physical activities (1); aerobic exercises (3); Basketball (1); Cycling or treadmill (1); Extreme activities (1); Treadmill (6); Walking and jogging (1); Bicycling (1); Unclear (2)	22 studies	England (1); France (1); Netherlands (1); USA (1); Iran (1); Brazil (8); Turkey (2); Portugal (1); China (2); South Africa (1); Australia (1); Egypt (2)	Systematic review and meta‐analysis	RCT(22)	Experimental = 474 Control = 458	Jadad score and Cochrane
Erik, 2020	Between August 1, 2012 and April 3, 2019	European Respiratory Journal	Cycling, jogging and aerobics (1); Unspecified aerobic exercise training (3); Treadmill training, education and breathing exercises (3); Jogging/Walking (2); Gym‐membership and intermittent personal training sessions and a diet intervention (1); Indoor cycling (1)	11 studies	NA	Systematic review and meta‐analysis	RCT(11)	Experimental = 270 Control = 273	Cochrane
Qiaoyu, 2022	Up to 1 November 1, 2021	Journal of Asthma and Allergy	Aerobic exercise (10); Free choice by child patients (3); Bicycle (3); Combined exercise (2); Resistance exercise (1)	18 studies	UK (1); Netherlands (1); France (1); Turkey( 1); Portugal (1); China (7); Australia (1); Saudi Arabia (1); Ireland (2); Denmark (1); Spain (1)	Meta‐analysis	RCT(18)	Experimental = 498 Control = 498	Cochrane
Sirpa, 2012	Between 1980 and June 2011	Eur J Epidemiol	Swimming (1); Aerobic exercise (3)	4 studies	Brazil (1); Serbia (1); England (1); Denmark (1)	Systematic review and meta‐analysis	RCT(4)	Experimental = 123 Control = 94	NA
YiRan, 2021	Up to April 2021	Evidence‐Based Complementary and Alternative Medicine	Aerobic training (6); Physical therapy based on exercise (1); Lung rehabilitation training program based on exercise (1); Exercise prescription (4); Combined exercise (1); Fixed treadmill exercise (1); Cycling (2); Indoor interval training (1); anaerobic exercise (4); Swimming (3)	22 studies	NA	Meta‐analysis	RCT(22)	Experimental = 703 Control = 653	Cochrane
Shengqiao, 2022	Up to October 2021	Annals of Translational Medicine	Aerobic exercises (4); Treadmill training (3); Unspecified (3); Walking (2); Gym and intermittent personal training sessions (1); Indoor cycling (1); Exercise training (3); Physical activity (1)	18 studies	UK (2); Iran (1); Brazil (7); Australian (2); Denmark (1); Kuwait (1); South Africa (2); US (1); Germany (1)	Systematic review and meta‐analysis	RCT(18)	Experimental = 544 Control = 529	Cochrane
Carson, 2013	Up to January 2013	Cochrane	Running (1); Aerobic exercises (9); Cycling (1); Physical training (2); Swimming(7)	20 studies	France (4); USA (2); Scotland (1); Brazil (6); Iran (1); Japan (1); Portugal (1); UK (1); Australia (1); Netherlands (1); China (1)	Systematic review and meta‐analysis	RCT(20)	Experimental = 382 Control = 374	Cochrane

In the 11 articles we included, a total of 162 studies revealed a relationship between physical activity and asthma. Beggs S [22] included eight swimming studies involving 262 participants and showed that swimming training was well tolerated by children and adolescents with stable asthma and enhanced lung function (moderate‐intensity evidence) and cardiorespiratory fitness (high‐intensity evidence). Hadassah et al. [23] included in a review of nine studies also showed that swimming significantly improved expiratory force expiration in one second (L) and expiratory lung capacity (%). However, in a review that included swimming and aerobic exercise, it was shown that regular physical activity improved physical fitness in adult asthmatics, but the effect on lung function was inconclusive [19]. Philipp [24] included 17 studies including 599 subjects, including swimming, running, aerobics, cycling, and other exercise modalities, and the results showed a significant improvement in FEV1. Xionghui's [18] meta‐analysis included 14 randomized controlled studies incorporating cycling, kickball, basketball, tai chi, swimming, set‐up exercises, gym strength training, jumping rope, and jogging, and showed that physical activity‐assisted therapy (the experimental group) resulted in a significant increase in FEV1 and FVC relative to the conventional treatment group. In six other reviews or meta‐analyses [21, 25, 26, 27, 28, 29] incorporating multiple exercise modalities, the results all suggested a substantial advantage of physical activity in improving FEV1, FVC, and VO2max in patients with asthma, although some of these had only a very low level of evidence. Table 2 shows the relationship between physical exercise and QoL, VO2max, and lung function in patients with asthma, respectively. Results from a total of 42 independent studies, of which seven were related to QoL, five to exercise capacity, and the remaining 30 to lung function.

**TABLE 2:** The relationship between physical exercises and QoL, VO2max, and lung function.

First author, year	Outcome indicators	No. of included studies	Type of study	Effects model	MA metric	Estimates	95%CI	Test for overall effect (*p* ‐value)	I2% (*p* ‐value)	Egger or Begg's test (*p* ‐value)	Publication bias and small‐study effect
Philipp, 2013	QoL	2	RCTs	Fixed	MD	8.9	[8.18,9.61]	< 0.001	59(0.12)	NA	NA
Xinggui, 2020	QoL	5(children)	RCTs	Random	WMD	0.81	[0.32,1.3]	< 0.001	72.1(0.006)	NA	NA
Xinggui, 2020	QoL	2(adults)	RCTs	Random	WMD	0.2	[0.07,0.32]	0.002	0(0.73)	NA	NA
Erik,2020	QoL	7	RCTs	Random	SMD	−0.48	[−0.81,−0.16]	0.004	45(0.09)	NA	NA
Qiaoyu,2022	QoL	9	RCTs	Random	MD	0.84	[−0.29,1.97]	0.15	100(< 0.001)	NA	NA
YiRan, 2021	QoL	12	RCTs	Random	SMD	1.28	[0.6,1.95]	0.0002	94(< 0.001)	Only funnel plot (N)	No publication bias
Shengqiao, 2022	QoL	10	RCTs	Random	SMD	−0.8	[−1.30,−0.31]	NA	86.9(< 0.001)	Only funnel plot (N)	No publication bias
Beggs S,2013	VO2max	3	RCTs	Fixed	MD	−7	[−14.57,0.57]	0.07	NA	NA	NA
Philipp,2013	VO2max	7	RCTs	Fixed	MD	4.06	[3.02,5.10]	< 0.001	38(0.14)	NA	NA
Sirpa,2012	VO2max	2	RCTs	Fixed	WMD	3.66	[3.44,3.88]	NA	98.7(< 0.001)	NA	NA
Shengqiao,2022	VO2max	5	RCTs	Random	MD	1.18	[0.87,1.48]	NA	17(0.31)	0.117	No publication bias
Carson,2013	VO2max	8	RCTs	Fixed	MD	4.92	[3.98,5.87]	< 0.001	44	NA	NA
Beggs S,2013	FEV1	4	RCTs	Fixed	MD	0.1	[−0.00,0.20]	0.062	32(0.22)	NA	NA
Beggs S,2013	FEV1%Pred	4	RCTs	Fixed	MD	8.07	[3.59, 12.54]	0.0004	38(0.19)	NA	NA
Beggs S,2013	FVC	4	RCTs	Random	MD	0.1	[−0.07,0.26]	0.24	57(0.07)	NA	NA
Beggs S,2013	FVC%	5	RCTs	Random	MD	3.85	[−0.58,8.28]	0.089	61(0.04)	NA	NA
Beggs S,2013	PEF	2	RCTs	Random	MD	62.07	[22.84,101.30]	0.0019	59(0.12)	NA	NA
Hadassah, 2021	FEV1	3	RCTs	Fixed	MD	0.22	[0.04,0.40]	0.01	0(0.59)	NA	NA
Hadassah, 2021	FEV1%Pred	5	RCTs	Fixed	MD	2.88	[−0.99,6.75]	0.15	0(0.95)	NA	NA
Hadassah, 2021	FVC%	5	RCTs	Random	MD	7.18	[−0.15,14.51]	0.05	72(0.006)	NA	NA
Hadassah, 2021	PEF%	4	RCTs	Fixed	MD	4.4	[−4.04,12.84]	0.31	0(0.86)	NA	NA
Philipp,2013	FEV1	11	RCTs	Fixed	MD	0.09	[−0.00,0.17]	0.05	29(0.17)	Only funnel plot (N)	No publication bias
Philipp,2013	PEF	6	RCTs	Random	MD	0.45	[−0.16,1.07]	0.15	81(< 0.001)	NA	NA
Xionghui, 2022	FEV1	13	RCTs	Random	SMD	−0.93	[−1.40,–0.45]	NA	91.6(< 0.001)	> 0.05	No publication bias
Xionghui, 2022	FVC	13	RCTs	Random	SMD	−0.77	[−1.23,−0.31]	0.001	91.5(< 0.001)	> 0.05	No publication bias
Xionghui, 2022	PEF	9	RCTs	Random	SMD	−0.6	[−1.03,−0.18]	0.006	86.9(< 0.001)	NA	NA
Xinggui,2020	FEV1	12	RCTs	Fixed	WMD	0.12	[0.05,0.20]	0.011	10.2(0.345)	0.663	No publication bias
Xinggui,2020	FEV1%Pred	11	RCTs	Fixed	WMD	0.68	[−0.64, 2.01]	0.808	36(0.111)	0.413	No publication bias
Xinggui,2020	FVC	10	RCTs	Fixed	WMD	0.18	[0.09,0.27]	0.003	0(0.817)	0.546	No publication bias
Xinggui,2020	FVC%	6	RCTs	Fixed	WMD	4.3	[0.88,7.72]	0.014	3.9(0.392)	NA	NA
Xinggui,2020	PEF	6	RCTs	Random	WMD	0.66	[0.24,1.09]	0.002	87.3(0)	NA	NA
Erik,2020	FEV1	10	RCTs	Random	SMD	−0.36	[−0.72,0.00]	0.05	69(0.0007)	NA	NA
Qiaoyu,2022	FEV1%Pred	18	RCTs	Random	MD	4.81	[1.57,8.05]	0.004	81(< 0.001)	NA	NA
Sirpa,2012	FEV1	3	RCTs	Fixed	WMD	0.07	[−0.27,0.41]	NA	0(0.768)	NA	NA
YiRan, 2021	FEV1%Pred	18	RCTs	Fixed	SMD	0.11	[−0.01,0.24]	0.07	19(0.22)	Only funnel plot (N)	No publication bias
YiRan, 2021	FVC%	15	RCTs	Fixed	SMD	0.27	[0.13,0.40]	< 0.001	36(0.08)	Only funnel plot (N)	No publication bias
YiRan, 2021	PEF%	11	RCTs	Random	MD	4.53	[1.27,7.80]	0.007	72(< 0.001)	Only funnel plot (N)	No publication bias
Shengqiao, 2022	FEV1	10	RCTs	Random	MD	0.24	[−0.13,0.60]	NA	81.4(< 0.001)	0.802	No publication bias
Shengqiao, 2022	FEV1%Pred	8	RCTs	Random	MD	0.47	[0.03,0.9]	NA	74.9(< 0.001)	0.741	No publication bias
Shengqiao, 2022	FVC	8	RCTs	Random	MD	−0.02	[−0.7,0.65]	NA	93.5(< 0.05)	0.842	No publication bias
Shengqiao, 2022	FVC%	5	RCTs	Random	MD	0.39	[−0.05,0.82]	NA	67.9(< 0.001)	0.141	No publication bias
Carson,2013	FEV1	9	RCTs	Fixed	MD	0	[−0.10,0.10]	0.99	45(0.07)	NA	NA

### 3.2 QoL

Table 3 summarizes the relative effects and their corresponding 95% CIs, as well as the quality of evidence, for the correlation between physical exercise and QoL in asthma. As shown in Table 2, a total of seven studies were included in the survey. The results of the study by Shengqiao et al. [28] demonstrated that physical activity had a significant advantage in improving the QoL of patients with asthma compared to the control group [SMD = −0.8,(−1.30, −0.31)], and the quality of evidence was rated as moderate. This can also be demonstrated by Xinggui et al.'s four studies [21, 24, 25, 27], but the quality of the evidence was either low or very low.

**TABLE 3:** AMSTAR2 and GRADE classification of the evidence for the correlation between physical exercise and asthma.

Summary of findings					Certainty assessment (degradation factor)					Certainty assessment (escalation factors)			Importance	Grade	AMSTAR2
First author, Year	Study design	No. of included studies	Outcome	Relative effect(95%CI)	Risk of bias	Inconsistency	Indirectness	Imprecision	Publication bias	Large effect	Plausible confounding	Dose response gradient			
Philipp, 2013	RCTs	2	QoL	MD 8.9 [8.18,9.61]	Serious^c^	Serious^a^	Not serious	Not serious	Strongly suspected	Yes	No	No	5	Very low	Critically low
Xinggui, 2020	RCTs	5 (children)	QoL	WMD 0.81 [0.32,1.3]	Serious^c^	Serious^a^	Not serious	Not serious	Strongly suspected	Yes	No	No	7	Low	Critically low
Xinggui, 2020	RCTs	2 (adults)	QoL	WMD 0.2 [0.07,0.32]	Serious^c^	Not serious	Not serious	Not serious	Strongly suspected	No	No	No	7	Low	Critically low
Erik, 2020	RCTs	7	QoL	SMD −0.48 [−0.81,−0.16]	Serious^c^	Not serious	Not serious	Not serious	Strongly suspected	No	No	No	7	Low	Critically low
Qiaoyu, 2022	RCTs	9	QoL	MD 0.84 [−0.29,1.97]	Not serious	Serious^a^	Not serious	Serious^b^	Strongly suspected	No	No	No	5	Very low	Critically low
YiRan, 2021	RCTs	12	QoL	SMD 1.28 [0.6,1.95]	Not serious	Serious^a^	Not serious	Serious^b^	Undetected	No	No	No	7	Low	Critically low
Shengqiao,2022	RCTs	10	QoL	SMD −0.8 [−1.30,‐0.31]	Not serious	Serious^a^	Not serious	Not serious	Undetected	No	No	No	7	Moderate	Critically low
Begg's S, 2013	RCTs	3	VO2max	MD −7 [−14.57, 0.57]	Not serious	Serious^a^	Not serious	Serious^b^	Strongly suspected	No	No	No	5	Very low	Low
Philipp, 2013	RCTs	7	VO2max	MD 4.06 [3.02,5.10]	Serious^c^	Not serious	Not serious	Not serious	Strongly suspected	Yes	No	No	7	Low	Critically low
Sirpa, 2012	RCTs	6	VO2max	WMD 3.66 [3.44,3.88]	Serious^c^	Serious^a^	Not serious	Not serious	Strongly suspected	No	No	No	5	Very low	Critically low
Shengqiao,2022	RCTs	5	VO2max	MD 1.18 [0.87,1.48]	Not serious	Not serious	Not serious	Not serious	Undetected	No	No	No	7	High	Critically low
Carson, 2013	RCTs	8	VO2max	MD 4.92 [3.98,5.87]	Not serious	Not serious	Not serious	Not serious	Strongly suspected	Yes	No	No	8	High	Low
Beggs S, 2013	RCTs	4	FEV1	MD 0.1 [−0.00, 0.20]	Not serious	Not serious	Not serious	Serious^b^	Strongly suspected	No	No	No	6	Low	Low
Beggs S, 2013	RCTs	4	FEV1%Pred	MD 8.07 [3.59, 12.54]	Not serious	Not serious	Not serious	Not serious	Strongly suspected	No	No	No	8	Moderate	Low
Beggs S, 2013	RCTs	4	FVC	MD 0.1 [−0.07, 0.26]	Not serious	Serious^a^	Not serious	Serious^b^	Strongly suspected	No	No	No	5	Very low	Low
Beggs S, 2013	RCTs	5	FVC%	MD 3.85 [−0.58, 8.28]	Not serious	Serious^a^	Not serious	Serious^b^	Strongly suspected	No	No	No	5	Very low	Low
Beggs S, 2013	RCTs	2	PEF	MD 62.07 [22.84, 101.30]	Not serious	Serious^a^	Not serious	Serious^b^	Strongly suspected	No	No	No	6	Very low	Low
Hadassah, 2021	RCTs	3	FEV1	MD 0.22 [0.04, 0.40]	Serious^c^	Not serious	Not serious	Serious^b^	Strongly suspected	No	No	No	6	Very low	Critically low
Hadassah, 2021	RCTs	5	FEV1%Pred	MD 2.88 [−0.99, 6.75]	Serious^c^	Not serious	Not serious	Serious^b^	Strongly suspected	No	No	No	5	Very low	Critically low
Hadassah, 2021	RCTs	5	FVC%	MD 7.18 [−0.15,14.51]	Serious^c^	Serious^a^	Not serious	Serious^b^	Strongly suspected	No	No	No	5	Very low	Critically low
Hadassah, 2021	RCTs	4	PEF%	MD 4.4 [−4.04,12.84]	Serious^c^	Not serious	Not serious	Serious^b^	Strongly suspected	No	No	No	5	Very low	Critically low
Philipp, 2013	RCTs	11	FEV1	MD 0.09 [−0.00, 0.17]	Serious^c^	Not serious	Not serious	Not serious	Undetected	No	No	No	7	Moderate	Critically low
Philipp, 2013	RCTs	6	PEF	MD 0.45 [−0.16,1.07]	Serious^c^	Serious^a^	Not serious	Serious^b^	Strongly suspected	No	No	No	5	Very low	Critically low
Xionghui, 2022	RCTs	13	FEV1	SMD −0.93 [−1.40,–0.45]	Serious^c^	Serious^a^	Not serious	Serious^b^	Undetected	No	No	No	5	Very low	Critically low
Xionghui, 2022	RCTs	13	FVC	SMD −0.77 [−1.23,−0.31]	Serious^c^	Serious^a^	Not serious	Serious^b^	Undetected	No	No	No	6	Very low	Critically low
Xionghui, 2022	RCTs	9	PEF	SMD −0.6 [−1.03,−0.18]	Serious^c^	Serious^a^	Not serious	Serious^b^	Strongly suspected	No	No	No	6	Very low	Critically low
Xinggui, 2020	RCTs	12	FEV1	WMD 0.12 [0.05, 0.20]	Serious^c^	Not serious	Not serious	Serious^b^	Undetected	No	No	No	7	Low	Critically low
Xinggui, 2020	RCTs	11	FEV1%Pred	WMD 0.68 [−0.64, 2.01]	Serious^c^	Serious^a^	Not serious	Not serious	Undetected	No	No	No	6	Low	Critically low
Xinggui, 2020	RCTs	10	FVC	WMD 0.18 [0.09,0.27]	Serious^c^	Not serious	Not serious	Serious^b^	Undetected	No	No	No	7	Low	Critically low
Xinggui, 2020	RCTs	6	FVC%	WMD 4.3 [0.88,7.72]	Serious^c^	Not serious	Not serious	Serious^b^	Strongly suspected	No	No	No	6	Very low	Critically low
Xinggui, 2020	RCTs	6	PEF	WMD 0.66 [0.24,1.09]	Serious^c^	Serious^a^	Not serious	Serious^b^	Strongly suspected	No	No	No	6	Very low	Critically low
Erik,2020	RCTs	10	FEV1	SMD −0.36 [−0.72,0.00]	Serious^c^	Serious^a^	Not serious	Not serious	Strongly suspected	No	No	No	5	Very low	Critically low
Qiaoyu, 2022	RCTs	18	FEV1%Pred	MD 4.81 [1.57, 8.05]	Not serious	Serious^a^	Not serious	Not serious	Strongly suspected	No	No	No	7	Low	Critically low
Sirpa,2012	RCTs	3	FEV1	WMD 0.07 [−0.27, 0.41]	Serious^c^	Not serious	Not serious	Serious^b^	Strongly suspected	No	No	No	5	Very low	Critically low
YiRan, 2021	RCTs	18	FEV1%Pred	SMD 0.11 [−0.01, 0.24]	Not serious	Not serious	Not serious	Serious^b^	Undetected	No	No	No	7	Moderate	Critically low
YiRan, 2021	RCTs	15	FVC%	SMD 0.27 [0.13,0.40]	Not serious	Not serious	Not serious	Not serious	Undetected	Yes	No	No	8	High	Critically low
YiRan, 2021	RCTs	11	PEF%	MD 4.53 [1.27,7.80]	Not serious	Serious^a^	Not serious	Serious^b^	Undetected	No	No	No	7	Low	Critically low
Shengqiao, 2022	RCTs	10	FEV1	MD 0.24 [−0.13, 0.60]	Not serious	Serious^a^	Not serious	Serious^b^	Undetected	No	No	No	6	Low	Critically low
Shengqiao, 2022	RCTs	8	FEV1%Pred	MD 0.47 [0.03, 0.9]	Not serious	Serious^a^	Not serious	Serious^b^	Undetected	No	No	No	6	Low	Critically low
Shengqiao, 2022	RCTs	8	FVC	MD −0.02 [−0.7, 0.65]	Not serious	Serious^a^	Not serious	Serious^b^	Undetected	No	No	No	5	Very low	Critically low
Shengqiao, 2022	RCTs	5	FVC%	MD 0.39 [−0.05,0.82]	Not serious	Serious^a^	Not serious	Serious^b^	Undetected	No	No	No	5	Very low	Critically low
Carson, 2013	RCTs	9	FEV1	MD0 [−0.10, 0.10]	Not serious	Serious^a^	Serious^a^	Not serious	Strongly suspected	No	No	No	5	Low	Low

^a^ Conclusions significant heterogeneity was reported.

^b^ The credible interval contains invalid values and the credible interval does not exclude significant benefits or harms.

^c^ Failure to adequately control for confounding.

### 3.3 VO2max

VO2max is the maximum oxygen consumption achieved during maximal exercise. It reflects the functional capacity of the three major systems of circulation, respiration, and exercise, which are closely related to the body's physical activity [31]. According to Table 3, physical exercise significantly improved VO2max [MD = 4.92,(3.98, 5.87)] in patients with asthma, and the quality of evidence was rated as advanced [29].

### 3.4 Pulmonary Function

As Table 3, the relative effect of the correlation between physical exercise and lung function in asthmatics and its corresponding 95% CI, as well as the quality of the evidence, were assessed. There were 30 independent outcomes, and one was rated as high‐level evidence. Physical activity was effective in improving FVC% in patients with asthma [SMD = 0.27, (0.13, 0.40)] [27]. Physical activity also improved FEV1%Pred [MD = 8.07, (3.59, 12.54)] in patients with asthma [22], and the quality of evidence was rated as moderate. Physical activity was similarly associated with improved FEV1, FVC, PEF, and PEF% in patients with asthma, but their evidence was rated as low or very low.

### 3.5 Heterogeneity, Publication Bias and Small Study Effect

Of all the items we summarized (all entries in Tables 2), 10 exhibited low heterogeneity (I^2^ < 25%), 19 showed moderate to high heterogeneity (25% < I^2^ < 75%), and 12 exhibited very high heterogeneity (I^2^ > 75%). In addition, one item failed to mention heterogeneity. The quality of the evidence is decreased for evidence with considerable heterogeneity (*p* < 0.05). This comprehensive review reviewed publication bias and the influence of small studies in meta‐analyses using the Egger's (or Begg's) test. Five research in the 11 meta‐analyses did not disclose any substantial publication bias, and the remaining six studies did not quantify publication bias (Tables 2).

### 3.6 Methodological Quality

The methodological quality scores for the 11 meta‐analyses that were part of this umbrella review are shown in Table 4. Of the 11 meta‐analyses, only two Cochrane reviews [22, 29] were rated as moderate quality, with the remaining nine meta‐analyses rated as very low quality. Overall, the major flaws of the critically low‐quality meta‐analyses fell short of items 2 and 7 on the AMSTAR2 scale. In other words, they did not register the protocol before conducting the meta‐analysis, nor did they provide a list of excluded studies or justify the exclusion.

**TABLE 4:** Methodological quality of the systematic review and meta‐analyses were assessed using the AMSTAR2 scale.

Study	Q1	Q2*	Q3	Q4*	Q5	Q6	Q7*	Q8	Q9*	Q10	Q11*	Q12	Q13*	Q14	Q15*	Q16	AMSTAR‐2 overall quality
Beggs	Y	Y	N	Y	Y	Y	Y	Y	Y	N	Y	Y	Y	Y	Y	Y	Moderate
Hadassah	Y	N	N	Y	Y	Y	Y	Y	Y	N	Y	Y	Y	N	N	Y	Critically low
Philipp	Y	N	N	PY	Y	Y	Y	Y	PY	N	Y	Y	N	N	Y	Y	Critically low
Xionghui	Y	N	N	PY	Y	N	N	PY	PY	N	Y	Y	Y	Y	Y	Y	Critically low
Xinggui	Y	N	N	PY	Y	Y	N	PY	Y	N	Y	Y	Y	Y	Y	Y	Critically low
Erik	Y	Y	N	Y	Y	N	N	Y	Y	N	Y	Y	Y	Y	N	Y	Critically low
Qiaoyu	Y	N	N	Y	Y	N	N	PY	Y	N	Y	Y	N	Y	N	Y	Critically low
Sirpa	Y	N	N	N	Y	Y	N	PY	N	N	Y	N	N	N	N	Y	Critically low
YiRan	Y	N	N	Y	Y	Y	N	PY	Y	N	Y	Y	Y	Y	Y	Y	Critically low
Shengqiao	Y	N	N	Y	Y	Y	N	PY	Y	N	Y	Y	Y	Y	Y	Y	Critically low
Carson	Y	Y	N	Y	Y	Y	Y	Y	Y	N	Y	Y	Y	Y	Y	Y	Moderate

*Note:* AMSTAR‐2 items: Q1: Did the research questions and inclusion criteria for the review include the components of PICO? Q2: Did the report of the review contain an explicit statement that the review methods were established prior to the conduct of the review, and did the report justify any significant deviations from the protocol? Q3: Did the review authors explain their selection of the study designs for inclusion in the review? Q4: Did the review authors use a comprehensive Literature search strategy? Q5: Did the review authors perform study selection in duplicate? Q6: Did the review authors perform data extraction in duplicate? Q7: Did the review authors provide a list of excluded studies and justify the exclusions? Q8: Did the review authors describe the included studies in adequate detail? Q9: Did the review authors use a satisfactory technique for assessing the risk of bias (RoB) in individual studies that were included in the review? Q10: Did the review authors report on the sources of funding for the studies included in the review? Q11: If meta‐analysis was performed, did the review authors use appropriate methods for statistical combination of results? Q12: If meta‐analysis was performed, did the review authors assess the potential impact of RoB in individual studies on the results of the meta‐analysis or other evidence synthesis? Q13: Did the review authors account for RoB in primary studies when interpreting/discussing the results of the review? Q14: Did the review authors provide a satisfactory explanation for, and discussion of, any heterogeneity observed in the results of the review? Q15: If they performed quantitative synthesis, did the review authors carry out an adequate investigation of publication bias (small study bias) and discuss its likely impact on the results of the review? Q16: Did the review authors report any potential sources of conflict of interest, including any funding they received for conducting the review?

## 4 Discussion

In recent years, the prevalence of asthma has increased. The prevention and treatment of asthma should be based on principles of long‐term, standardized, and individualized management [32]. According to the BTS [33] Guidelines on pulmonary rehabilitation, patients with chronic respiratory diseases should be actively engaged in pulmonary rehabilitation. However, the lack of clinical evidence and reports of exercise‐induced asthma symptoms makes physical activity very much frowned upon by most patients with asthma [34]. To date, there is considerable debate as to whether physical activity can be used as a complementary alternative therapy for pulmonary rehabilitation in patients with asthma and whether it is effective in improving patients' QoL and exercise capacity. Our study, which incorporates data from earlier systematic reviews and meta‐analyses, offers a thorough summary of the effects of physical activity on QoL, exercise capacity, and lung function in patients with asthma in order to advance understanding of the relationship between physical activity and asthma.

In this review of systematic evaluations of physical activity and asthma, we included 11 eligible studies with a total of 42 independent findings. The results showed that physical activity was significantly better than the control group in improving the QoL and exercise capacity of patients with asthma, as well as in improving the patients' lung function indices, such as FVC% and FEV1%Pred. However, there was no moderate or high evidence that exercise was associated with improvement in the indices of FEV1, FVC, PEF, and PEF%. In previous studies, pulmonary rehabilitation based on respiratory muscle exercises (e.g., respiratory muscle exercises in yoga exercises) has been shown to have a definite positive effect on patients with asthma [35], we did not include pulmonary rehabilitation exercises based on respiratory muscle training in our study.

Improvement of pulmonary function in asthmatics by physical activity may stem from a number of factors. Airway inflammation is one of the important components of asthma, and exercise combined with medication improves oxidative stress thereby improving lung function. It has been noted that patients with asthma who have undergone physical activity have reduced serum C‐reactive protein (CRP), exhaled nitric oxide (FeNO), and sputum eosinophil counts (EOS) [36]. A study by France‐Pinto et al. showed that patients who implemented an exercise prescription for 12 weeks had decreased airway hyper reactivity (AHR) and significant decreases in interleukin‐6 (IL‐6), and monocyte chemotactic proteins‐1 (MCP‐1) compared to controls [37]. In addition, it was also found in experiments on animal models of asthma that aerobic training reduced inflammatory cell infiltration, decreased EOS migration and nuclear factor‐κB (NF‐κB) expression by inducing an increase in the expression of anti‐inflammatory cytokines, while decreasing the expression of Th2 cytokine‐β, transforming growth factor‐β (TGF‐β), leukocyte chemokines (eotaxin and RANTES), vascular endothelial growth factor (VEGF), and adhesion molecules (ICAM‐1, VCAM‐1) pro‐inflammatory factors expression and decreased AHR and inflammatory factor levels in asthmatic mice [38]. Furthermore, collagen deposition, increased mucus secretion and airway remodeling are thought to reduce airway patency and compliance. It was demonstrated that exercise intervention reduced airway collagen deposition, smooth muscle thickness and epithelial mucus in OVA‐sensitized mice. Certainly, further research on the mechanisms by which exercise improves lung function and QoL in patients with asthma is still warranted [39].

AMSTAR2 and GRADE were the assessment instruments utilized in this umbrella review. The methodological quality of the meta‐analyses included in this umbrella review was evaluated by AMSTAR2. Research questions, inclusion of standard PICO elements, system review plan, study design type, literature search strategy, literature screening, data extraction, exclusion of specific details from the literature, assessment of bias risk, assessment of the rationality of statistical analysis, assessment of the accuracy of result interpretation, and assessment of financial support and conflict of interest are among the main aspects of evaluation that are covered [14]. GRADE is used in systematic reviews and meta‐analyses to assess the quality of the evidence, or how much the predicted outcome's authenticity can be guaranteed [15, 16]. We classified the quality of evidence for the systematic evaluation as high, moderate, low, and very low by looking at three upgrading factors—large effect, dose–response gradient, and plausible confounding—and five downgrading factors—risk of bias, indirectness, inconsistency, imprecision, and publication bias [15, 16].

One of the strongest standards of evidence in evidence‐based medicine is the Umbrella Review [40]. It summarizes the data from many sources and critically evaluates all published meta‐analyses and systematic reviews on medical subjects [41]. The publication of systematic reviews and meta‐analyses of study findings has increased dramatically in recent years. While this has closed a significant evidence vacuum in clinical decision‐making, it has also complicated physicians' ability to make medical decisions. Umbrella reviews have therefore gained more clout in the field of evidence‐based medicine.

This research has certain restrictions. Initially, the structure of umbrella reviews compels us to incorporate systematic reviews or meta‐analyses in lieu of primary research, potentially hindering our ability to scrutinize the latest advancements pertinent to asthma management. Second, there may be publication bias because there were few original studies for some of the relationships in this investigation that were included in the relevant meta‐analyses. Third, the great diversity of interventions across studies and the fact that most studies did not analyze subgroups made it difficult to obtain precise data. Fourth, because there was a small amount of raw data that we could not access during the data extraction process, we had to downgrade it accordingly in the final evaluation of the quality level of the evidence.

## 5 Conclusion

The complex information that is currently available about how physical activity affects asthma is compiled in this study. The findings demonstrated that QoL of patients with asthma and the ability to exercise were significantly enhanced by physical activity. Additionally, physical activity improves pulmonary function in patients with asthma, although the overall quality of evidence is insufficient. In the future, more high‐quality prospective studies are required.

## Author Contributions

Conception and design: Fuchun Huang, Shuang Yang, Mingxuan Ma, Hua Liu. Administrative support: Hua Liu. Collection and assembly of data: Fuchun Huang, Shuang Yang, Mingxuan Ma. Data analysis and interpretation: Fuchun Huang, Shuang Yang, Mingxuan Ma, Jialin Zhang. Manuscript writing: Fuchun Huang, Shuang Yang. Final approval of manuscript: All authors.

## Ethics Statement

This study did not involve any human or animal experiments, and all data were derived from previously published meta‐analyses. All data are open source and can be obtained from the corresponding author.

## Conflicts of Interest

The authors declare no conflicts of interest.

## Supporting information


**Table S1**Search terms utilized in the umbrella review (Search date up to Dec 30, 2022).

**Table S2** Full text screening of excluded studies and a list of reasons for their exclusion.

## Data Availability

The data that supports the findings of this study are available in the supplementary material of this article.
