# Supplementary material for: Effects of Physical Exercises in Asthma: An Umbrella Review of Systematic Review and Meta‐Analysis
Source: Clin Respir J. 2025 Apr 17;19(4):e70075. doi: 10.1111/crj.70075 (PMC12004085; doi:10.1111/crj.70075)
Supplement: Supplementary file 1 — Table S1Search terms utilized in the umbrella review (Search date up to Dec 30, 2022). [file CRJ-19-e70075-s002.docx]

**Supplemental Table S1.** Search terms utilized in the umbrella review (Search date up to Dec 30 2022)

| Terms 1 | Terms 2 | Terms 3 | Database | Restrictions | Results |
| --- | --- | --- | --- | --- | --- |
| endurance OR strength OR exercise OR training OR sport OR physical activity OR run OR cycle OR swim OR gymnastic OR aerobic exercise OR anaerobic exercise | asthma OR wheezing OR bronchial asthma | meta-analysis OR systematic review OR systematic overview | PUBMED | All field | 421 |
| TS=(endurance OR strength OR exercise OR training OR sport OR physical activity OR run OR cycle OR swim OR gymnastic OR aerobic exercise OR anaerobic exercise) | TS=(asthma OR wheezing OR bronchial asthma) | TS=(meta-analysis OR systematic review OR systematic overview) | WOS | Topic | 789 |
| endurance OR strength OR exercise OR training OR sport OR physical activity OR run OR cycle OR swim OR gymnastic OR aerobic exercise OR anaerobic exercise | asthma OR wheezing OR bronchial asthma | meta-analysis OR systematic review OR systematic overview | Cochrane | Cochrane  Reviews | 44 |
