# Supplementary material for: Effects of Physical Exercises in Asthma: An Umbrella Review of Systematic Review and Meta‐Analysis
Source: Clin Respir J. 2025 Apr 17;19(4):e70075. doi: 10.1111/crj.70075 (PMC12004085; doi:10.1111/crj.70075)
Supplement: Supplementary file 2 — Table S2 Full text screening of excluded studies and a list of reasons for their exclusion. [file CRJ-19-e70075-s001.docx]

**Table S2:** Full text screening of excluded studies and a list of reasons for their exclusion

| Serial number | 1st Author | Year | Title | Reason for Exclusion |
| --- | --- | --- | --- | --- |
| 1 | Lene Lochte | 2016 | Childhood asthma and physical activity: a systematic review with meta-analysis and Graphic Appraisal Tool for Epidemiology assessment | No RCT |
| 2 | Marianne Eijkemans | 2012 | Physical Activity and Asthma: A Systematic Review and Meta-Analysis | No RCT |
| 3 | Vera HM Wanrooij | 2013 | Exercise training in children with asthma: a systematic review | No meta-analysis |
| 4 | Margaret M | 2021 | A Systematic Review of the Effect of Physical Activity on Asthma Outcomes | No meta-analysis |
| 5 | Alison Crosbie | 2012 | The Effect of Physical Training in Children With Asthma on Pulmonary Function, Aerobic Capacity and Health-Related Quality of Life: A Systematic Review of Randomized Control Trials | No meta-analysis |
| 6 | DIANA R | 2012 | Exercise-Related Quality of Life in Subjects with Asthma: A Systematic Review | No meta-analysis |
| 7 | Jason E | 2019 | The impact of exercise on asthma | No meta-analysis |
| 8 | Rebecca F | 2022 | Increasing physical activity in severe asthma: a systematic review and meta-analysis | Breathing training |
| 9 | Smita Pakhale | 2013 | Effect of physical training on airway inflammation in bronchial asthma: a systematic review | Lack of outcome indicators of interest |
| 10 | Sarah R | 2022 | Exercise Training Programs Improve Cardiorespiratory and Functional Fitness in Adults With Asthma | Lack of outcome indicators of interest |
